# Circulating oestradiol determines liver lipid deposition in rats fed standard diets partially unbalanced with higher lipid or protein proportions

**DOI:** 10.1017/S0007114521004505

**Published:** 2022-10-28

**Authors:** Laia Oliva, Marià Alemany, José-Antonio Fernández-López, Xavier Remesar

**Affiliations:** 1 Department of Biochemistry and Molecular Biomedicine, Faculty of Biology, University of Barcelona, Barcelona, Catalonia 08028, Spain; 2 Institute of Biomedicine (IBUB), University of Barcelona, Barcelona, Spain; 3 CIBER OBN, Research Web, Barcelona, Spain

**Keywords:** Energy partition, Liver lipid deposition, High-fat diet, High-protein diet, Oestrogens

## Abstract

The ingestion of excess lipids often produces the accumulation of liver fat. The modulation of diet energy partition affects this process and other metabolic responses, and oestrogens and androgens are implied in this process. Ten-week-old male and female rats were fed with either standard rat chow (SD), SD enriched with coconut oil (high-fat diet, HF), SD enriched with protein (high-protein diet, HP) or a ‘cafeteria’ diet (CAF) for 1 month. HF and CAF diets provided the same lipid-derived percentage of energy (40 %), HP diet protein energy derived was twice (40 %) that of the SD. Animals were killed under anaesthesia and samples of blood and liver were obtained. Hepatic lipid content showed sex-related differences: TAG accumulation tended to increase in HF and CAF fed males. Cholesterol content was higher only in the CAF males. Plasma oestradiol in HF and HP males was higher than in CAF. Circulating cholesterol was inversely correlated with plasma oestradiol. These changes agreed with the differences in the expression of some enzymes related to lipid and energy metabolism, such as fatty acid synthetase or phosphoglycolate phosphatase. Oestrogen protective effects extend to males with ‘normal’ diets, that is, not unbalanced by either lipid or protein, but this protection was not enough against the CAF diet. Oestradiol seems to actively modulate the liver core of 2C-3C partition of energy substrates, regulating cholesterol deposition and lactate production.

The data obtained using high-fat diets almost invariably result in increased body and liver weights^(1)^, including a variety of alterations in homoeostatic markers, especially overall increased fat deposition, but also, markedly in liver, altering glucose and lipid metabolism^(2,3)^. Furthermore, the incidence of dietary fatty acids in oestrogen synthesis^(4)^, and the role of oestrogens controlling the main metabolic pathways related to lipid metabolism^(5)^, points to the influence of these hormones on the fate of the diet energy substrates, depending in part on their fatty acid content.

On the other hand, diets with high-protein content have been promoted, especially in humans, to maintain or increase muscle mass^(6)^, although they have also been applied to the treatment of obesity and related diseases^(7,8)^. However, their actual metabolic effects on energy partition and on general regulation of metabolism are largely unknown^(9,10)^, a situation compounded by the lack of information on amino acid diverse catabolism pathways and by the factors that regulate their utilisation under normal feeding conditions. In addition, there are marked sex differences in energy management, since females tend to show lower energy efficiency than males^(11)^. Since oestrogens increase the sensitivity to insulin^(12)^ and androgens also contribute to glycaemic homoeostasis^(13)^, supported by abundant literature we can assume that the implication of both groups of hormones in energy partition is significant^(14)^.

Despite the liver key strategic position (and role) in handling the substrates derived from the diet, the mechanisms of energy partition have been poorly studied both under normalcy and excessive feeding. We expected that diet lipid and/or protein content should affect liver metabolism, altering its ability for lipid oxidation and deposition. We also assumed that the role of oestrogens and/or androgens would help explain the different responses observed in males and females. In this experiment, we used controls which fed essentially the same diet components than the groups with an added burden of protein or lipid. Furthermore, we added a classical and proven obesogenic diet type, cafeteria, which we expected could behave as a ‘wild card’ to help explain the way energy partition was modulated by ‘naturally released’ oestrogen in both female and male rats.

## Experimental methods

### Animals and experimental setup

All animal handling procedures and the experimental set-up were carried out in accordance with the animal handling guidelines of the European, Spanish and Catalan Authorities. The Committee on Animal Experimentation of the University of Barcelona authorised the specific procedures used (# DAAM 6911).

Ten-week-old female (initial weight 233 (se 8) g) and male (initial weight 364 (se 12) g) Wistar rats (Janvier) were used (*n* 52), fed *ad libitum* for 30 d. The animals were randomly divided into four groups (*n* 6–8 each) for each sex: rat chow (standard diet, the SD group), a SD mixed with coconut oil (high-fat diet, the HF group), a simplified cafeteria diet (the CAF group)^(15)^ or a SD mixed with proteins (high-protein diet, the HP group). All animals had free access to water, and they were housed (in same-sex pairs) in a controlled environment (lights on from 08.00 to 20.00 hours, temperature 21·5–22·5°C and 50–60 % humidity). Body weight and cage food consumption were recorded daily. The calculation of ingested food in rats fed CAF diet was done as previously described by weighing the differences in food offered and debris left^(16)^ and correcting for food drying weight loss.

### Diets

Table 1 shows the composition of the diets used. The SD (Teklad 2014, Teklad diets) contained 19 % of digestible energy derived from protein, 13 % from lipids and 67 % from carbohydrates (including 10 % from oligosaccharides). This diet essentially contained plant-derived foods. Diets were prepared as previously described^(17,18)^. Thus, HF diet was prepared by the addition of refined coconut oil (Escuder SL) to coarsely ground standard chow and contained 14 % of digestible energy derived from protein, 37 % from lipids and 49 % from carbohydrates (no oligosaccharides). The standard chow pellets, plain cookies spread with liver pâté, bacon, water and milk, to which sucrose and a mineral and vitamin supplement was added, formed the simplified CAF diet^(15)^. All components were kept fresh (i.e. renewed daily). From the analysis of diet components and the ingested items, we calculated that ingested CAF diet contained 40 % of energy derived from lipids, 12 % from protein and 49 % from carbohydrates (24 % from oligosaccharides). The HP diet was prepared by the addition of equal dry proportions of casein and gelatine (Escuder SL) to the ground standard chow; in this case, the energy derived from proteins was 40 %, and 12 % that derived from fat; the energy from carbohydrates was 48 % (in these supplemented diets, containing only polysaccharides, as in the SD controls). Gelatin was used as a glue to maintain the pellet structure. In addition, collagen (from which gelatin is an industrial derivative) is in most animals and foods an abundant protein. Thus, the combination casein–gelatin results in a protein supplement closer to the expected mean protein composition of animal-derived foods.

**Table 1.** Diet composition and diet components (Mean values and standard errors)

	Standard diet (SD)	High-fat diet (HF)	Cafeteria diet* (CAF)		High-protein diet (HP)
	%	%	Mean	se	%
Crude energy content (kJ/g)¶	16·5	18·8	12·4	0·2	17·4
Digestible energy content (kJ/g)#	12·1	14·6	12·0	0·1	12·4
Gross composition					
Protein	14·3	11·6	7·96	0·4	28·7
Lipid	4·0	13·4	11·6	0·9	3·89
Carbohydrate	48·0	39·1	31·7	0·5	33·7
Fibre	18·0	14·6	2·79	0·2	12·9
Ashes	4·7	3·8	2·79	0·1	3·30
Moisture	6·9	12·4	47·2	1·2	12·3
Cholesterol	<0·001	<0·001	0·428		<0·001
Food components of diet (g/kg) components (g/kg)					
Chow pellet	1000	901	113	8	804
Coconut oil		99			
Gelatin					100
Casein					87
Bacon (^CHO^800 mg/kg)			123	1·1	
Cookies (plain)			209	15	
Liver pâté (^CHO^2·25 g/kg)			126	13	
Cows’ milk (full fat; ^CHO^140 mg/l)			335	25	
Sunflower oil					9
Sucrose			101	12	
Energy derived from nutrients					
Protein	19·3	14·5	11·7	0·4	40·4
Lipid	12·5	36·8	39·5	0·9	6·0
Carbohydrate	67·1	48·6	48·5	0·5	47·7
Sugars (as % of carbohydrate) carbohydrates)	<1	<1	24·1	0·5	<1
Lipid/protein ratio	0·625	2·53	3·4	0·2	0·15

* Data obtained from the food consumption data of the animals fed cafeteria diet (mean male values).

¶ Crude energy refers to the total energy equivalence of all diet’s components, and digestible energy# only to the energy derivable from digestible protein, lipids and carbohydrates, excluding fibre.

Intake differences between male and female animals were not statistically significant.

^CHO^Cholesterol content.

The absence of data represents the practical absence in the corresponding food component.

The SD, HF and HP diets were presented to the rats in the form of dry extruded pellets. Aversion tests to this diet gave negative results, not being different from control diet as indicated previously^(17)^. Animals did not show any distress signs during the procedure.

### Fatty acid analyses

Lipids from food samples were extracted overnight with the trichloromethane/methanol (2:1 v/v) and processed for fatty acid analysis as previously described^(18)^. Briefly, samples were suspended in 10 % boron trifluoride (Fluka) in methanol and were stored in the dark at 4°C for 12 h. Hexane and water were added. After mixing, the completely organic phase was extracted, filtered and dried. The residue was dissolved in hexane (Panreac) and the samples were analysed with a GC-MS system (QP2010 Shimadzu), using a SP-2560 Supelco column (Supelco). An extended methylated fatty acid mixture (Supelco FAME mix C4-C24) was used as the standard. Calculations were performed using the Shimadzu FASST for GC-MS software (version 2). Rates of recovery of lipids (and of fatty acid samples) were determined using internal standards of *bis*-C17:0 diacylglycerol (Sigma-Aldrich).

### Experimental procedure

After 30 d of treatment, at the beginning of a light cycle, the rats were anaesthetised with isoflurane, and blood was withdrawn with dry-heparinised syringes, through the exposed aorta until death by exsanguination. Plasma was obtained by centrifugation and kept at −20°C until processed. Liver was dissected and immediately frozen in liquid nitrogen and then weighed and stored. Rats had continuous access to food up to their euthanasia.

### Analytical procedures

Total N, lipid and energy content of diet components were analysed as previously described^(19)^. Plasma parameters were measured using standard commercial kits: urea was measured with kit #11537, total cholesterol with kit #11505 and TAG with kit #11528 (all from Biosystems). Lactate was measured with kit #1001330 (Spinreact) and non-esterified fatty acids with kit NEFA-HR (Wako); 3-hydroxybutyrate and acetoacetate were estimated with a ketone bodies kit (Biosentec). Glycerol was estimated with kit #F6428 (Sigma-Aldrich). Elisa kits EIA1559 and EIA2693 were used to determine testosterone and oestradiol (DRG International). Glucose in plasma was measured with a glucose oxidase kit #11504 (Biosystems) supplemented with mutarotase (490 nkat/ml of reagent) (Calzyme). Mutarotase was added to speed up epimerisation equilibrium of *α*- and *β*-d-glucose and thus facilitate the complete oxidation (i.e. measurement) of d-glucose by glucose oxidase^(20,21)^.

### Liver determinations

Samples of frozen liver (30–50 mg) were powdered under liquid N_2_. A mixture of trichloromethane-methanol solution (1 ml; 2:1 v/v) was added to the liver powder, shaken and left at room temperature for 1 h, with occasional shaking to complete lipid extraction. Water (200 µl) was added to the tubes, vortexed and centrifuged at 3000 × g during 5 min. The upper phase was discarded, and the organic phase was then dried with dry N at room temperature. The lipid pellet was dissolved in 2-methyl-2-propanol (60 µl) and Triton X-114-methanol (40 µl; 2:1 v/v) mix^(22)^. Liver TAG and cholesterol content were measured using the glycerol and cholesterol kits, respectively (Biosystems). Total protein was measured using the Lowry *et al.* method^(23)^.

### Gene expression analyses

Total tissue RNA was extracted from frozen samples (*ca*. 50 mg) using the Tripure reagent (Roche Applied Science) and was quantified in a ND-1000 spectrophotometer (Nanodrop Technologies). RNA samples were reverse transcribed using the MMLV RT (Promega) system and oligo-dT primers. Real-time PCR amplification was carried out using 10 μl amplification mixtures containing Power SYBR Green PCR Master Mix (Applied Biosystems), 10 ng of reverse-transcribed RNA and 300 nm primers. Reactions were run on an ABI PRISM 7900 HT detection system (Applied Biosystems) using a fluorescent threshold manually set to 0·15 for all runs. A semi-quantitative approach for the estimation of the concentration of specific gene mRNA per unit of tissue weight was used^(24)^. *Cyclophillin A (Ppia)* was used as the charge control gene. The data were expressed as the number of transcript copies per gram of liver protein, in order to obtain comparable data between the groups, given the uniformity of the samples in that aspect. The genes analysed and a list of primers used are presented in Complementary Table 1. These genes are related to lipid metabolism, lipid metabolism transcription factors or energy metabolism. Thus, *Pgp* (phosphoglycolate phosphatase) down-regulates the cell levels of glycerol-3-P; *Fasn (*fatty acid synthase*)* catalyses the synthesis of palmitate from acetyl CoA; *Hmgcs2* (3-hydroxymethyl-glutaryl-CoA synthase 2) catalyses the first reaction of ketogenesis and *CPT1α* (carnitine-palmitoyl-transferase 1) initiates the oxidation of fatty acids. On the other hand, *Cox4i1* (cytochrome C oxidase subunit4 isoform1) and *Uqcrc1* (ubiquinol-cytochrome C reductase core protein 1) are part of respiratory electron transport system. Finally, *Srebf2* (sterol regulatory element binding) and *Pparα* (peroxisome proliferator activated receptor α) are transcription factors which control cholesterol metabolism (*Srebf2*) and overall lipid *(Pparα).*

### Calculations

Statistical comparisons were performed using two-way ANOVA (sex and diet) and Bonferroni’s *post hoc* tests, using the Prism 5.0 programme (GraphPad Software). Differences obtained with ANOVA or the Bonferroni’s *post hoc* test were considered statistically significant when the *P* value was <0·05. Correlations between different parameters were determined by linear regression analysis using the same programme, applying a 95 % CI and the value of the Pearson correlation coefficient.

## Results

Table 1 describes the nutrient composition of the diets. The values for CAF diet were obtained from the actual consumption data and were like those previously described^(17,18)^ (which were used to design the lipid content of the HF diet). Diet energy contents, both crude (i.e. total) and digestible (i.e. that of nutrients potentially used for energy), were higher in the HF diet, since its energy density (kJ/g) was higher than those of SD, HP and CAF groups. The CAF diet had the lowest crude energy intake value because of its abundant food water (as milk) and despite a low fibre content, although its digestible energy was akin to that of the SD. Fat content of CAF and HF diets was similar and 3-fold higher than those of SD and HP diets. HP diet showed the highest proportion of protein, with a lipid content in the range of that of the SD. HF diet was the richest in C12:0 + C14:0 fatty acids, followed by the CAF diet.

Table 2 shows the diet’s fatty acid composition. SD and HP showed high levels of oleic and linoleic acids, whereas in the HF diet saturated acids predominate, especially lauric acid. CAF diet showed a high disparity between their components, being cookies the richest in SFA. However, the mean fatty acid intake resulted in the ingestion of 54 % SFA (22 % from lauric + myristic acids), 34 % of monounsaturated and 12 % of PUFA (Table 1).

**Table 2.** Diet’s fatty acid composition (mg/g)

			Cafeteria diet (CAF)				
	Standard diet (SD)	High-fat diet (HF)	Cookies	Milk	Pâté	Bacon	High-protein diet (HP)
Saturated							
C 10:0	0	4·29	6·4	0·34	0·23	0·88	0
C 12:0	0·21	37·8	44·2	0·37	0·47	0·49	0
C 14:0	0·34	12·2	14·9	1·7	2·69	3·59	0·09
C 16:0	6·91	14·2	24·7	5·72	19·7	72·2	5·59
C 18:0	0·51	2·25	14·2	2·42	20·7	26·1	0·76
C 20:0	0·06	0·07	1·62	0·14	2·76	3·04	0·07
C 22:0	2·47	0	2·54	0·27	5·07	4·76	2·15
Monounsaturated							
C 16:1	0	0	1·8	0·25	5	6·43	0
C 18:1	5·78	18·7	28·3	4·77	96·4	117	5·71
C 20:1	0·14	0·16	1·53	0·21	3·54	3·09	0·13
C 22:1	0	0	1·02	0·11	2·56	2·69	0
Polyunsaturated							
C 18:2	23·9	18·7	8·85	0·47	20·9	32·5	22·6
C 18:3	1·97	1·92	1·15	0·20	1·96	2·5	1·59
C 20:4	0	0	0·78	0·08	2·02	2·49	0

The 1-month energy intake of all chow pellet-derived groups was similar (8–9 MJ for males and 5–6 MJ for females, different from CAF-fed groups, which ingested almost twice the energy than the other groups)^(19)^. Therefore, changes in weight increase were similar for all groups, except for the highest values of CAF (Table 3). Another consequence of this different energy intake was that the weight of white adipose tissue (in the perigonadal, mesenteric and retroperitoneal locations) was equivalent in all groups, except in CAF (40 or 48 % higher in, respectively, males and females)^(19)^. In spite of these differences, liver weight showed only sex-related differences, not significantly affected by diet.

**Table 3.** Weight increase, liver weight, metabolite and hormone plasma values of rats after dietary treatment (Mean values with their standard errors)

	Males								Females								ANOVA
	SD diet		HF diet		CAF diet		HP diet		SD diet		HF diet		CAF diet		HP diet		
	Mean	se	Mean	se	Mean	se	Mean	se	Mean	se	Mean	se	Mean	se	Mean	se	
Weight increase (g)	79·1	8·2^a^	82·2	6·3^a^	126	3·2^b^	68·6	1·5^a^	39·5	4·3^a^	27·5	1·6^a^	73·6	6·9^b^	31·2	1·8^a^	D S
Final weight (g)	445	30^a^	436	12^a^	515	4·6^b^	444	45^a^	245	7·6^a^	262	2·8^a^	290	7·7^b^	255	8·1^a^	D S
Energy intake (MJ)	8·76	0·44^a^	8·43	0·21^a^	19·2	0·59^b^	7·90	1·01^a^	6·32	0·24^a^	5·98	0·91^a^	16·1	0·29^b^	5·41	0·12^a^	D S
Liver weight (g)	15·9	1·57^a,b^	13·1	0·33^a^	16·7	0·85^b^	15·1	0·15^a,b^	8·56	0·47^a^	8·14	0·19^a^	8·16	0·24^a^	7·66	0·37^a^	S
Glucose (mM)	9·85	0·31^a^	9·65	0·51^a^	10·5	0·7^a^	11·2	0·6^a^	10·9	0·7^a^	8·43	0·31^b^	10·4	0·6^a,b^	10·1	0·3^a,b^	D
Lactate (mM)	2·07	0·08^a^	4·71	0·49^b^	2·66	0·30^a^	4·40	0·31^b^	2·27	0·31^a^	4·01	0·28^b^	2·70	0·33^a,c^	3·70	0·29^b,c^	D
Glycerol (mM)	0·16	0·02^a^	0·34	0·06^a^	0·18	0·03^a^	0·35	0·06^a^	0·15	0·03^a^	0·21	0·01^a^	0·22	0·02^a^	0·21	0·01^a^	D S
Cholesterol (mM)	2·66	0·21^a^	1·65	0·08^b^	2·24	0·11^a^	1·61	0·07^b^	2·64	0·11^a^	1·58	0·21^b^	2·48	0·31^a^	1·36	0·04^b^	D
NEFA (mM)	0·32	0·05^a^	0·38	0·04^a^	0·55	0·08^a^	0·45	0·06^a^	0·32	0·05^a^	0·35	0·03^a^	0·44	0·07^a^	0·44	0·06^a^	D
TAG (mM)	1·34	0·04^a^	1·94	0·11^b,c^	1·96	0·18^b^	1·35	0·19^a,c^	1·01	0·09^a^	1·04	0·21^a^	0·98	0·08^a^	0·77	0·14^a^	D S
Cholesterol/TAG	1·97	0·13^a^	0·90	0·10^b^	1·59	0·15^a,c^	1·13	0·13^b,c^	2·71	0·33^a^	1·90	0·21^a^	2·82	0·29^a^	1·78	0·19^a^	D S
NEFA/TAG	0·29	0·05^a^	0·33	0·06^a^	0·24	0·03^ac^	0·33	0·04^a^	0·37	0·07^a^	0·49	0·08^a^	0·42	0·05^a^	0·57	0·11^a^	S
Urea (mm)	2·98	0·31^a,b^	3·55	0·11^a^	1·98	0·15^b^	6·62	0·35^c^	2·26	0·18^a,b^	3·43	0·31^a^	1·88	0·31^b^	6·76	0·43^c^	D
3OH butyrate (µm)	31·2	5·01^a^	48·9	4·51^a^	31·1	5·75^a^	47·2	5·50^a^	47·1	7·11^a^	62·4	11·1^a^	31·4	6·78^a^	60·6	11·1^a^	D S
Acetoacetate (µm)	190	43·5^a^	159	32·4^a^	128	15·9^a^	143	12·2^a^	145	58·5^a^	183	31·4^a^	202	49·4^a^	110	25·2^a^	
Oestradiol (ng/l)	29·1	5·24^a^	100	8·41^b^	35·9	3·82^a^	69·8	6·05^c^	57·2	4·58^a^	106	6·96^b^	59·7	6·07^a^	111	10·5^b^	D S
Testosterone (µg/l)	3·18	0·28^a^	3·51	0·44^a^	3·69	0·49^a^	2·96	0·19^a^	1·16	0·07^a^	1·72	0·08^b^	1·26	0·11^a^	1·47	0·08^a,b^	S
Testosterone/oestradiol	111	17·1^a^	36·9	6·94^b^	102	8·08^a^	41·5	5·55^b^	20·8	2·02^a^	16·1	1·27^a,b^	21·1	1·38^a^	13·2	2·11^b^	D S

Statistical analysis: two-way ANOVA significant *P*-values for diet (D), sex (S) or interaction (I): *P* < 0 05.

Statistical significance (Bonferroni’s *post hoc* test) between diets is represented by different superscript letters.

Animal and liver weights increased in parallel in all groups. The energy intake and plasma parameters are shown in Table 3. HF diets, compared with SD, showed higher plasma levels of lactate and oestradiol, and lower cholesterol, both in males and females. Female HF diets also showed lower glucose and higher testosterone than SD, whereas males showed higher plasma TAG. CAF diet only showed higher increases in males’ TAG when compared with SD. The HP diets induced, in both sexes, a marked increase in circulating urea, also in parallel to higher lactate and oestradiol. The HP rats showed lower cholesterol values than the SD controls. The effect of diet type was significant for all parameters analysed except for acetoacetate and testosterone, which remained unchanged. We found global differences between both sexes for glycerol, TAG, hydroxybutyrate, and, as expected, for oestradiol and testosterone.

**Fig. 1.**
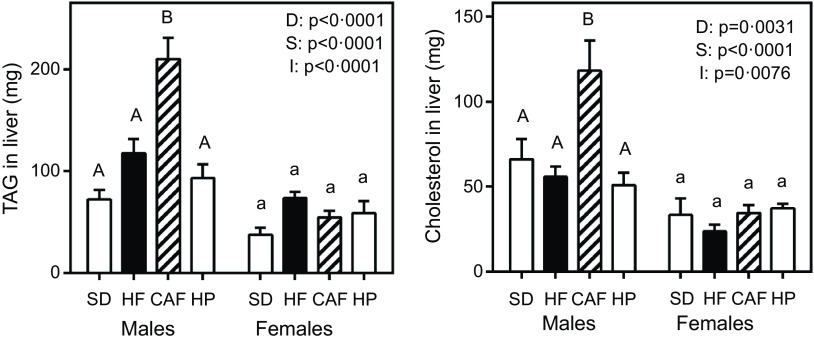
TAG and cholesterol liver content. Data are the mean with their standard error of the mean of six to eight animals per group. White bars: standard diet (SD); black bars: high-fat diet (HF); dashed bars: cafeteria diet (CAF) and lattice bars: high-protein diet (HP). Statistical differences between groups: two-way ANOVA (D, diet; S, sex; I, their interaction). Bonferroni’s *post hoc* test: different letters represent statistically significant differences between groups of the same sex.

Liver fat and cholesterol concentrations are presented in Fig. 1. Male rats fed CAF diet showed a significant 3-fold increase of TAG accumulation in their livers compared with the SD-fed animals. Cholesterol accumulation was also higher in male CAF group, but HF and HP liver cholesterol values were not different from those of SD. Female rats had lower liver cholesterol and TAG than the males, with no significant differences between the dietary groups.

In the males, significant correlations were found between liver TAG content and lipid intake (*P* = 0·0080) and between cholesterol content and lipid intake (*P* = 0·0050). However, the females did not show any significant correlation for these parameters. Testosterone levels did not correlate with liver TAG (*P* = 0·50) nor cholesterol (*P* = 0·09) content, irrespective of sex.

**Fig. 2.**
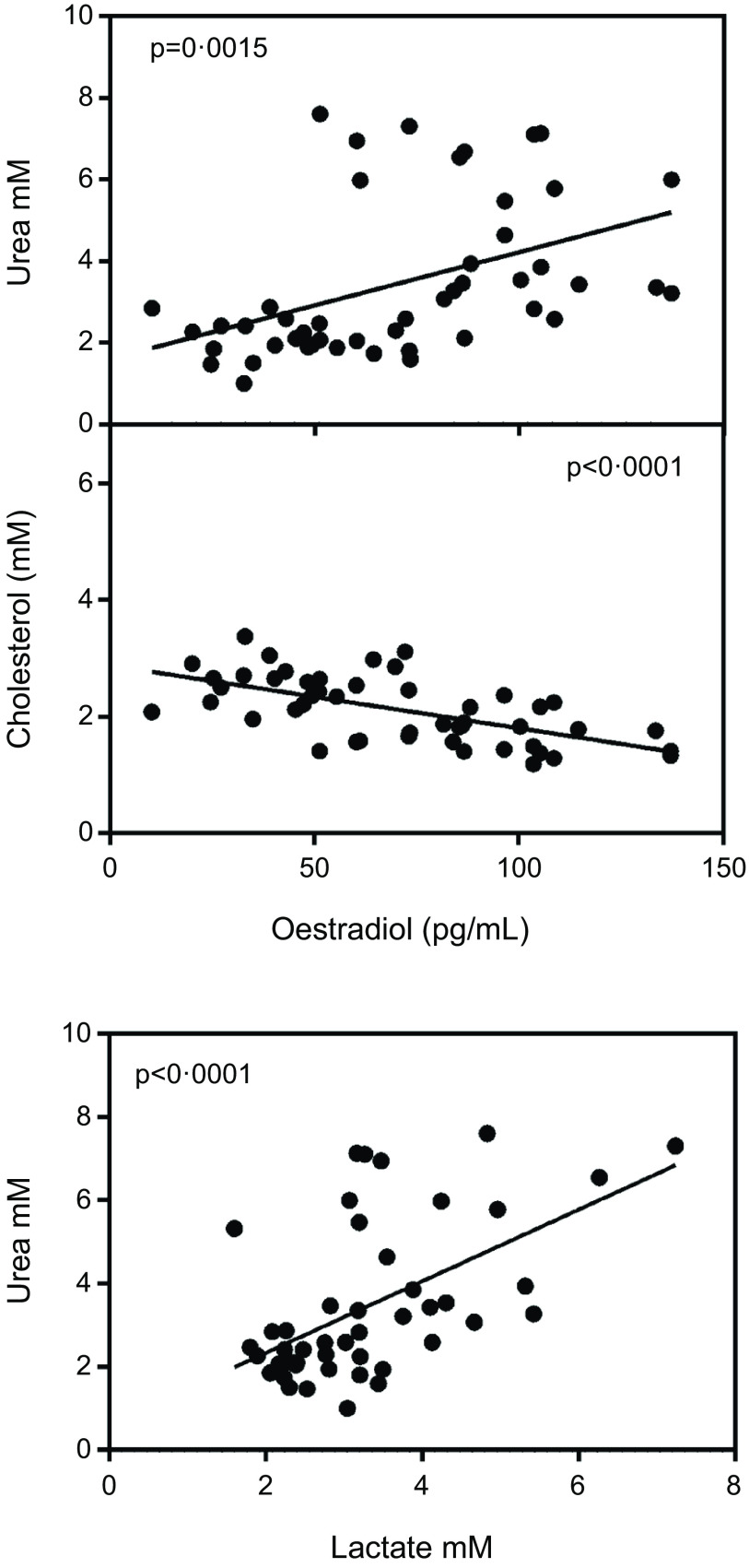
Correlation of plasma oestradiol *v*. plasma cholesterol and urea values and plasma lactate *v*. urea. Values of statistical significance of correlations are incorporated as *P* values.

Fig. 2 shows a significant inverse correlation between plasma oestradiol and plasma cholesterol levels. In addition, the figure shows that plasma urea was correlated with oestradiol and also with lactate. Conversely, testosterone levels were not correlated with cholesterol (*P* = 0·60), urea (*P* = 0·87) or lactate (*P* = 0·16), irrespective of sex.

**Fig. 3.**
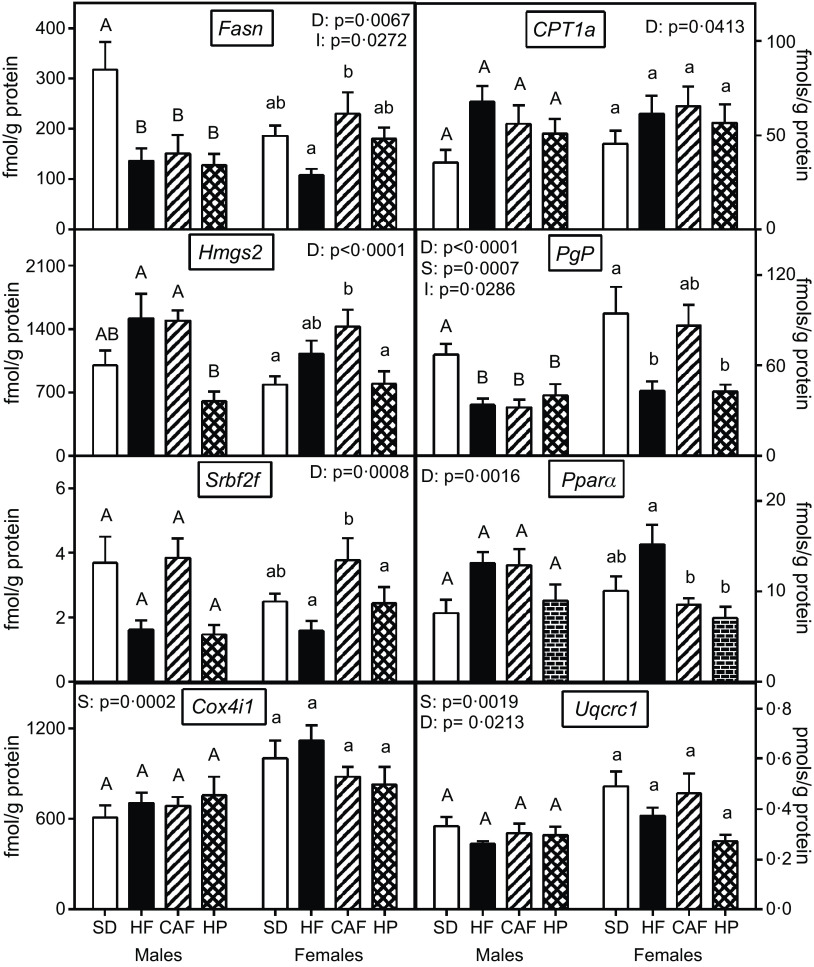
Liver expression of different enzymes or transcription factors: fatty acid synthase (*Fasn*), carnitine O-palmitoyl transferase 1 (*Cpt1a*), hydroxymethyl-glutaryl-CoA synthase 2 (*Hmgs2*), phosphoglycolate phosphatase (*PgP*), sterol regulatory element-binding protein 2 (*Srbf2f*), peroxixome proliferator activated receptor α (*Pparα)*, cytochrome C oxidase I (*Cox4i1*) and ubiquinol-cytochrome C reductase core protein 1 (*Uqcrc1*). Data are the mean with their standard error of the mean of six to eight animals per group. White bars: standard diet (SD); black bars: high-fat diet (HF); dashed bars: cafeteria diet (CAF) and lattice bars: high-protein diet (HP). Statistical differences between groups: two-way ANOVA (D, diet; S, sex; I, their interaction). Bonferroni’s *post hoc* test: different letters represent statistically significant differences between groups of the same sex.

Fig. 3 shows the variations in gene expression caused by dietary treatment. Thus, the decreases in *Fasn* expression in HF, CAF and HP groups in males contrast with the lack of changes in *CPT1a*. *PgP* expression followed a similar pattern than *Fasn*, including decreases in HF and HP groups in females. *Hmgs2* showed differences between HF and CAF in relation to HP males. Transcription factors showed different patterns, as *Srbf2f* showed a clear tendency to decrease in HF and HP expression in males, whereas *Pparα* showed differences, in HF females, with respect to CAF and HP diet groups. Significant sex-related differences were observed, however, for the expressions of *Pgp, Cox4i1* and *Uqcrc1*, females showing, in all cases, higher expression values than males.

## Discussion

As expected, the self-selected CAF diet induced a higher energy intake^(19)^ that caused higher weight increases than any of the other diets: the main cause for this change was an inordinately high accrual of adipose tissue^(19,25)^. It is interesting to note that the HF-treated rats did not increase their weight over that of the SD diet controls despite their high proportion of fat intake (the same as CAF), confirming that a high lipid intake *per se* is not associated with a disproportionate increase in fat reserves^(26)^. The observed increase in male liver lipid content can be a consequence of the surplus of energy derived from sucrose^(27)^, thus doubling the lipid content of the other groups. This result also suggests that CAF males were unable to export the excess TAG synthesised in the liver, despite their increased plasma levels, thus contributing to an incipient hepatic steatosis^(26)^. This pattern contrasts with that of females eating the CAF diet, which, despite their high fat intake, did not show hepatic lipid accumulation. We assume that this differential pattern may be a consequence of the different hormonal status and not simply energy accounting, since the females also have a lower energy efficiency than males^(11)^. The ubiquitous presence of oestrogens in females potentiates the oxidative capacity of mitochondria through enhanced cytochrome C oxidase activity^(28)^ and other paths and effects^(29)^. The higher liver expressions of *Cox4i* and *Uqrc1c* in all female groups, coupled with lower expression of factors implied in triacylglycerol synthesis, such as *PPARα* ^(30)^ attest to a lower energy efficiency than their male counterparts. The differences in liver lipid accumulation between CAF and HF groups are, probably, a direct consequence of the differences in oestradiol, with raised levels in the HF group, and of a higher cholesterol in CAF. In addition, oestrogens favour the decrease in lipogenesis^(31)^, and in rodents fed HF diets, facilitate the lipoprotein export of TAG, in part by increasing the expression of ApoA5^(32)^.

The high levels of oestradiol found in the HF group could be – at least in part – a consequence of the presence of high lauric acid^(33)^, which is known to increase the activity of aromatase^(4)^ (that converts the A ring of testosterone, dihydrotestosterone or 11b-hydroxytestosterone to oestrogens) and an active substrate to treat inflammation^(34)^. However, the supplementation of diet with coconut oil reduces lipogenesis in our model (decreases the expressions of *Fasn* and *Srbf2f*) and tends to increase the oxidation of fatty acids, mediated in part by *Pparα* ^(35)^ and *Cpt1.* These changes should be a consequence of increased oestradiol levels, induced by the action of lauric acids on aromatase.

Nevertheless, the marked differences observed in the handling of fat by female rats compared with the males suggest that the known influence of medium-chain fatty acids could be in part countered by the low overall unsaturation of the fat. This may be the case of HF diet, since saturated fats have been found to increase fat deposition^(36)^. It is important to note the radical differences in fat handling pattern shown by females and males, since the female liver did not accumulate TAG irrespective of the dietary amount or type of fatty acids ingested and reinforces the assumed role of oestrogen as a main causative effect of the differential lipid handling efficiency. This assumption also agreed with the differences observed on plasma metabolites and, ultimately, with the higher ability of females to handle excess energy^(11)^. The postulated female trading of this higher oxidative capability for a lower deposition helps safeguard their core energy partition homoeostasis. In fact, this spend thrift characteristic represents a net advantage to face situations in which the excess energy can compromise survival, as is the case with the metabolic syndrome^(2)^. This fact was accomplished despite the absence of differences with respect to the males for ketogenesis, as indicated by the expression of *Hmgs2.*

The liver expression of the oestrogen receptor (*α*) is higher in females than in males^(11)^. Oestrogen receptors (*α*) are implied in limiting liver fat deposition, regulated through membrane receptor signalling^(37)^. In females fed high-energy diets, TAG and cholesterol are stored in lipid droplets, metabolically active organelles, where a low level of Cidec/Fsp27*β* expression induces a lower presence of CIDEC/FSP27 protein^(38)^, pointing towards a hormonal regulation (oestrogens) of their turnover. However, this fact does not seem to be sex related because HF and HP males also show relatively high plasma levels of oestradiol. The effect of oestradiol lowering liver lipid content has also been attributed to sequestration of *Srbef1* on the membrane^(37)^. Our results also indicate a substantial decrease in the expression of *Srbef2*, suggesting its implication in the decrease of cholesterol synthesis in the male HF group. This may also help explain the inverse correlation found between cholesterol and oestradiol levels. Our results agree with the observed decrease in liver cholesterol synthesis caused by HF diets or by cholesterol-supplemented diets^(39)^.

The experimental data show a direct effect of HP diets on oestradiol levels not previously described to our knowledge. However, this increase may help explain the effects on body lipids of hyperproteic diets^(40)^ unburdened by lipid or sugars. This may be a direct consequence of the anaplerotic effects of 5C, 4C fragments formed in the oxidation of the hydrocarbon skeleton of amino acids^(14)^, plus a significant provision of 3C (and 2C), which helps spare glucose when needed but speeds up the oxidation of 2C (acetyl-CoA and consequently fatty acids). In addition, amino acids and oestradiol^(41)^ help potentiate the secretion, stability and effectiveness of insulin in maintaining glycaemic homoeostasis.

In our experimental model, testosterone does not seem to play a direct role in the regulation of dietary lipid deposition in the liver, since we did not found any correlation with lipid metabolism (except for being fundamental for the synthesis of oestradiol^(42)^). Because of this function, and the direct protagonism of oestradiol, we have not found effects on other deposition-related parameters, including circulating cholesterol. Furthermore, the direct correlation between urea and oestradiol confirms that the lipid-catabolic role of oestrogens is not only directed to spare glucose for oxidation and to increase lactate production but also favouring the use of excess amino acids for energy^(14)^.

A protective role of oestrogens against obesogenic diets has been described in mice^(43)^, where a dimorphic activity of glucocorticoid metabolising enzyme has been postulated to justify this different sexual trend^(44)^. On the other hand, the high plasma levels of lactate showed by HF and HP groups, in a metabolically normoglycaemic condition, may be a consequence of the stimulated activity of phosphofructokinase^(45)^, and then, 3C metabolite (pyruvate, lactate, glycerol and amino acids that produce the former metabolites) sparing, in front of 2C metabolite (acetyl-CoA) use^(14)^, a shift elicited by high oestradiol. In consequence, plasma oestradiol levels (within the physiological range for both sexes) do change liver metabolism by deeply modifying energy partition, limiting cholesterol deposition and increasing 3C lactate production from glucose, rather than its complete oxidation in the liver via acetyl-CoA (2C) in the mitochondria.

The postulated effect of oestrogen, which was based largely on correlations, is not an exclusive peculiarity of female rats, since males also show a modulation of their oestradiol levels in relation to diet and lipid (energy) handling. Long time ago, we already observed anti-obesity effects of oestrone fatty esters in the face of high-energy diets, irrespective of sex^(46)^, devoid of patent oestrogenic effects despite prolonged treatments^(47)^. It is well known that oestrogens favour the utilisation of excess body fat^(48)^, whereas the regulation of sex steroids in plasma via sex hormone binding globulin in humans seems solely limited (in practical terms) to testosterone^(49)^, in a context fully disconnected from oestrogens. The presence of active 17-hydroxysteroid dehydrogenases in liver^(50)^ suggests a direct proximity between the regulatory agent and the regulated paths. The marked absence of additional studies on the direct implication of oestradiol on the hub of energy partition and its regulation makes further assumptions more difficult, except that oestradiol is known to directly affect the oxidative function of mitochondria^(29)^, thus being able to speed up the oxidation of acetyl-CoA, a critical point for the removal (despite the overall inefficiency of the process) of unneeded acetyl-CoA and indirectly preventing its incorporation to the lipogenic pathway.

The role of oestrogens preventing lipid deposition (especially in the liver) operates not only for the females but also extends to males on HF and HP, regardless of the large difference between both diets. This uniformity of action suggests that oestradiol plays a more general role than usually assumed in the context of dietary energy partition, largely irrespective of sex and influencing also the handling of cholesterol and the conversion of glucose to lactate (or other 3C fragments) or its use to provide 2C fragments for energy, ketogenesis of lipogenesis.

## Supplementary material

For supplementary material accompanying this paper visit https://doi.org/10.1017/S0007114521004505. click here to view supplementary material
