# Supplementary material for: Circulating oestradiol determines liver lipid deposition in rats fed standard diets partially unbalanced with higher lipid or protein proportions
Source: Br J Nutr. 2021 Nov 15;128(8):1499–508. doi: 10.1017/S0007114521004505 (PMC9557166; doi:10.1017/S0007114521004505)
Supplement: Supplementary file 1 [file S0007114521004505sup001.docx]

**Supplementary Table 1.** Primer sequences used for gene expression analysis.

| **gene** | **protein** | **directionnn** | **Sequences** | **Bp** |
| --- | --- | --- | --- | --- |
| **Pgp** | Phosphoglycolate phosphatase | 5' > | CCTGGACACAGACATCCTCCT | 100 |
|  |  | > 3' | TTCCTGATTGCTCTTCACATCC |  |
| **Fasn** | Fatty acid synthase | 5' > | CCCGTTGGAGGTGTCTTCA | 117 |
|  |  | > 3' | AAGGTTCAGGGTGCCATTGT |  |
| **Cox 4i1** | Cytochrome C oxidase subunit 4 isoform 1 | 5' > | AGATGTAGACACCCGAGCCT | 172 |
|  |  | > 3' | TGTTAGGCCCCCTACTGTGA |  |
| **Hmgcs2** | Hydroxymethyl-glutaryl-CoA synthase 2, mitochondrial | 5' > | CAACCTCTTCCCAGGCACTT | 108 |
|  |  | > 3' | CCGGGGAATGGTTGTATGGA |  |
| \| ***CPT1a*** \| *Carnitine O-palmitoyltransferase 1, liver isoform* \| \| --- \| --- \| | Carnitine O-palmitoyl-transferase 1, liver isoform | 5' > | CCGCTCATGGTCAACAGCA | 105 |
|  |  | > 3' | CAGCAGTATGGCGTGGATGG |  |
| ***Srebf2*** | Sterol regulatory element-binding protein 2 | 5' > | ACCGTTTAGCAGCCACAGCA | 121 |
|  |  | > 3' | CCACAACCCTGACCAACACC |  |
| **Pparα** | Peroxisome proliferator activated receptor alpha | 5’ > | GCACAATCCCCTCCTGCAAC | 124 |
|  |  | > 3’ | TTCAATGCCCTCGAACTGGA |  |
| **Uqcrc1** | Ubiquinol-cytochrome C reductase Core Protein 1 | 5’ > | TCGCAGCCTCCTGACTTATG | 78 |
|  |  | > 3’ | ATCTGGGCATCCACCTCCT |  |
| **PPIA** | Peptidylprolyl isomerase A (cyclophillin A) [housekeeping gene] | 5' > | CTGAGCACTGGGGAGAAAGGA | 87 |
|  |  | > 3' | GAAGTCACCACCCTGGCACA |  |
